# Electrochemical Corrosion Properties and Protective Performance of Coatings Electrodeposited from Deep Eutectic Solvent-Based Electrolytes: A Review

**DOI:** 10.3390/ma18030558

**Published:** 2025-01-26

**Authors:** Vyacheslav S. Protsenko

**Affiliations:** Ukrainian State University of Science and Technologies, Lazaryan St. 2, 49010 Dnipro, Ukraine; vprotseko7@gmail.com

**Keywords:** corrosion behavior, protective properties, electrochemical deposition, deep eutectic solvents

## Abstract

The application of deep eutectic solvents (DESs) as an innovative class of environmentally friendly liquid media represents a significant advancement in materials science, especially for the development and enhancement of structural materials. Among the promising applications, DESs are particularly attractive for the electrodeposition of corrosion-resistant coatings. It is established that corrosion-resistant and protective coatings, including those based on metals, alloys, and composite materials, can be synthesized using both traditional aqueous electrolytes and non-aqueous systems, such as organic solvents and ionic liquids. The integration of DESs in electroplating introduces a unique capacity for precise control over microstructure, chemical composition, and morphology, thereby improving the electrochemical corrosion resistance and protective performance of coatings. This review focuses on the electrodeposition of corrosion-resistant and protective coatings from DES-based electrolytes, emphasizing their environmental, technological, and economic benefits relative to traditional aqueous and organic solvent systems. Detailed descriptions are provided for the electrodeposition processes of coatings based on zinc, nickel, and chromium from DES-based baths. The corrosion–electrochemical behavior and protective characteristics of the resulting coatings are thoroughly analyzed, highlighting the potential and future directions for developing anti-corrosion and protective coatings using DES-assisted electroplating techniques.

## 1. Introduction

Metal-based coatings are extensively applied to metal substrates for corrosion protection due to their excellent resistance properties [1]. Among the different methods, electrodeposited coatings are widely favored in the industry for their low cost, strong adhesion, and straightforward application process. In electroplating, the coating is formed by depositing material onto the substrate through electrolysis, which enables advantages like uniform coverage, controlled thickness, and ease of use [2,3]. Additionally, the electrodeposition process allows for the production of nanocrystalline coatings, which often exhibit superior corrosion resistance and protective qualities [4,5]. The corrosion behavior of electrodeposited coatings is a critical factor that determines their overall protective effectiveness. By modifying the electroplating bath composition and adjusting electrolysis conditions, both the corrosion resistance of the coating itself and its protective function for the substrate metal can be effectively tailored.

Various factors affect the corrosion performance of electrodeposited coatings, including the coating’s composition, deposition parameters, and substrate surface preparation [1,2]. The composition of the coating material influences corrosion performance by modifying the anodic and cathodic polarization characteristics. Deposition conditions also play a role by shaping the coating’s thickness, morphology, and crystalline structure. Additionally, substrate preparation affects adhesion and barrier properties by eliminating contaminants and creating a clean, textured surface appropriate for coating application. Electrochemical deposition techniques, such as pulse and pulse-reverse electrolysis, further allow for precise tuning of the coating’s microstructure, composition, and corrosion-resistant properties [6].

Electrodeposited coatings serve as a protective barrier for metal substrates by preventing corrosive agents from reaching the surface. The effectiveness of this barrier depends on factors like coating thickness, surface smoothness, and defect presence. Additionally, electrodeposited coatings can offer sacrificial anodic protection, where the coating material corrodes in place of the substrate. This sacrificial behavior is determined by the anodic polarization properties of the coating material.

The protective effectiveness of electrodeposits can be improved by altering the composition, morphology, and crystalline structure of the coatings. By incorporating various alloying elements, the composition of the coating material can be adjusted to enhance its corrosion resistance. Additionally, modifications of the deposition parameters, such as current density, temperature, and pH, can significantly affect the surface morphology of a coating. Such alterations may result in the development of nanocrystalline coatings, which typically demonstrate superior corrosion resistance and protective capabilities owing to their distinctive structural characteristics.

As stated above, the deposition of a “foreign” metal coating on a metal substrate creates a protective barrier that prevents the substrate from interacting with corrosive environments. Consequently, one might expect that the corrosion behavior of coated samples is solely influenced by the corrosion characteristics of the electrodeposited metal. However, a notable aspect of the electrochemical corrosion of electrodeposited coatings, which significantly affects their protective performance, is the presence of pores and cracks. In aggressive environments, these imperfections can give rise to galvanic corrosion cells. Within these galvanic microcells, depending on the electrode potential ratios, either the coating metal or the substrate metal may function as the anode or the cathode. Based on this behavior, electrodeposited coatings composed of metals and their alloys can be categorized into two main groups [7], (a) anodic coatings and (b) cathodic coatings.

Anodic coatings consist of metals that are more electronegative than the underlying metal substrate. These coatings are typically known as sacrificial coatings. A common instance is the application of zinc on a steel surface. The standard electrode potentials for the electrochemical couples Zn(II)/Zn and Fe(II)/Fe are −0.76 V and −0.44 V, respectively. Therefore, if pores develop in the zinc coating, the zinc will serve as a sacrificial anode, while the steel substrate will be cathodically polarized, effectively preventing the electrochemical corrosion of the steel from a thermodynamic viewpoint.

Cathodic coatings are composed of metals that are more electropositive compared to the substrate metal. A typical example is a copper coating on steel, where the standard electrode potential for the Cu(II)/Cu couple is +0.34 V. In this scenario, substrate protection relies solely on the barrier properties of the coating. If the coating has pores, the substrate metal may undergo anodic dissolution in the resulting galvanic cells during electrochemical corrosion. However, there are instances where intentionally creating numerous tiny pores in the coating can be beneficial, as this promotes uniform corrosion of the base metal instead of localized corrosion. This approach can be advantageous for ensuring the long-term performance of products.

The electrodeposition of multilayer coatings is a method employed to improve the corrosion resistance and protective capabilities of coatings [8]. This technique often involves the application of an intermediate layer between the substrate and the noble coating, such as nickel–chromium layers. Typically, a bright nickel layer with a high sulfur content is deposited over a dull nickel layer, which renders it more noble than the steel substrate. Subsequently, a finishing layer of bright chromium is applied over the bright nickel layer. Such multilayer systems provide exceptional corrosion protection [7]. Another example of multilayer coatings is a reported electrodeposition process for a three-layer zinc–iron–chromium coating [9], where an intermediate iron layer was utilized to enhance the adhesion of chromium to zinc, leading to a coating with commendable protective properties.

Electrochemical deposition from deep eutectic solvents (DESs) has garnered growing interest as an alternative to conventional aqueous and organic solvents for depositing metal-based coatings [10]. While DESs are not a new class of ionic liquids, as they are eutectic mixtures that exhibit a temperature depression lower than that of an ideal mixture [11,12,13], only those DESs that exhibit ionic conductivity are typically used for metal electrodeposition. This is because ionic conductivity is essential for the electroplating process. As a result, in the literature on metal deposition from DESs, these solvents are often conditionally referred to as a new class of ionic liquids due to their ability to conduct electricity [10,11,12]. Furthermore, the components of DESs commonly used for electroplating are generally chosen for their low toxicity and biodegradability [11]. Common examples of DESs include “ethaline” (a eutectic mixture of choline chloride and ethylene glycol in a 1:2 molar ratio) and “reline” (a eutectic mixture of choline chloride and urea, also in a 1:2 molar ratio) [11]. The melting temperatures for these eutectic ionic liquids, as well as for their individual components, are provided in Table 1. It can be noted that these DESs remain liquid at temperatures near room temperature.

**Figure 1:**
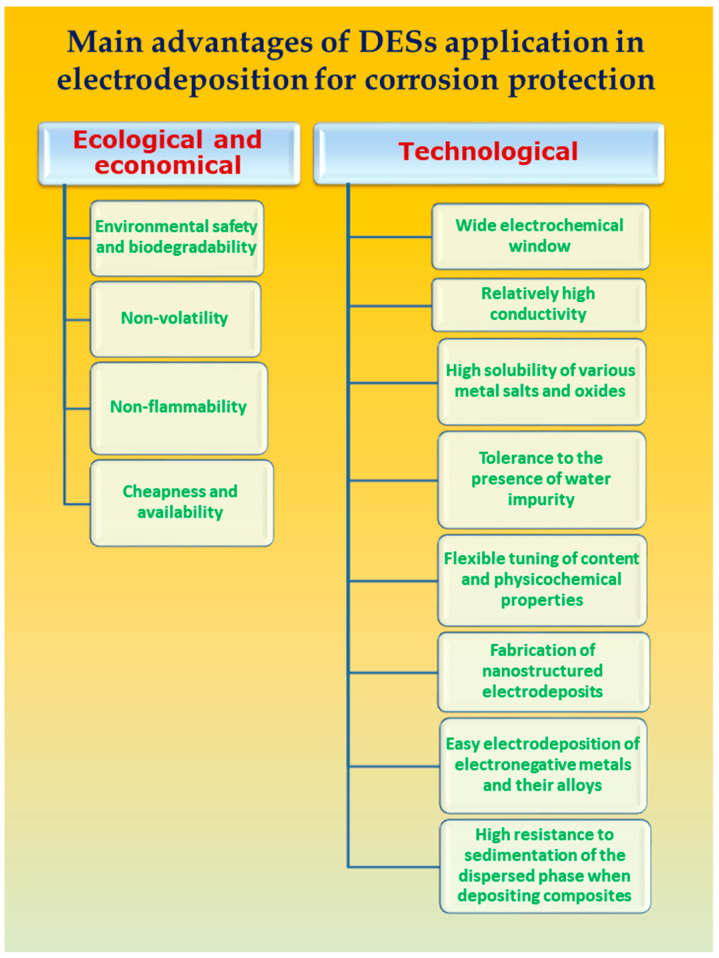
Main advantages of DES application in electrodeposition for corrosion protection.

DESs present a unique combination of advantages over traditional solvents (Figure 1), including low toxicity, minimal flammability, and high biodegradability [11,12]. This combination of properties sets DESs apart from aqueous solutions, organic solvents, and classical ionic liquids, making them particularly attractive for diverse applications. Notably, DESs can dissolve a broad spectrum of compounds, such as inorganic salts and organic molecules, providing remarkable flexibility as solvents, including for the electrodeposition of metal-based coatings.

Electrodeposition using DESs provides benefits like reduced toxicity, enhanced conductivity, and the capability to deposit metals at lower temperatures compared to conventional solvents [14,15,16]. Certain characteristics of DESs, including high viscosity and relatively low conductivity, can influence the electrodeposition process, particularly affecting the nucleation and growth phases of the coating material. In addition, the application of environmentally friendly DES-assisted plating baths opens up opportunities to produce nanomaterials with excellent service properties [17].

In recent years, numerous studies have explored the electrochemical corrosion behavior and protective properties of metal-based coatings deposited using DESs. It is important to note that both cathodic and anodic protective coatings can be electrodeposited from DES-based solutions. This review seeks to examine the key characteristics and unique aspects of the corrosion resistance and protective capabilities of certain metal-based coatings produced from DES-assisted electroplating solutions. Specifically, we will analyze the electrodeposition processes and corrosion resistance of coatings based on zinc, nickel, and chromium, as these metals represent some of the most essential and widely used types of corrosion-resistant protective coatings in industrial applications. The novelty of this study lies in the comprehensive evaluation of DES-based electrolytes as an innovative alternative to conventional aqueous and organic systems for electroplating. By focusing on the unique advantages of DESs, such as enhanced environmental compatibility, improved control over coating composition and microstructure, and energy efficiency, this review provides a new perspective on how DES-assisted electroplating can revolutionize the development of corrosion-resistant coatings with superior protective properties.

## 2. Electrochemical Corrosion Characteristics and Protective Performance of Coatings

### 2.1. Corrosion Resistance and Protective Properties of Zinc-Based Coatings

Zinc and its alloys are widely used as electrodeposited coatings in industries such as engineering, automotive, and aerospace to safeguard steel surfaces against corrosion [18]. While specific requirements for pure zinc coatings are not consistently outlined in the literature, their widespread use is primarily attributed to the well-established understanding of their anti-corrosion properties and cost-effectiveness.

Typically, Zn and its alloys are electrodeposited from aqueous solutions, including cyanide baths, alkaline cyanide-free baths, and acid chloride baths; however, these solutions are toxic and aggressive to a greater or lesser extent. Although these industrial methods are well established and readily available, they come with notable drawbacks, including hydrogen embrittlement, reduced current efficiency, and challenges related to wastewater management [19]. These, along with other limitations of aqueous zinc electroplating electrolytes, drive interest in developing alternative non-aqueous electrolytes, particularly those utilizing DESs [20].

The electrochemical deposition of zinc has been explored using electrolytes based on two types of eutectic mixtures, namely choline chloride with ethylene glycol (ethaline) [21,22,23] and choline chloride with urea (reline) [24,25]. Not all of these studies, however, have addressed the corrosion resistance and protective behavior of the deposited zinc layers. One notable exception is [25], in which zinc coatings were electroplated from an electrolyte containing 0.4 M zinc chloride and 0.1 M thiourea dissolved in reline. The deposition was conducted at 80 °C with a pulsed current setup involving a pulse current density of 2.5 mA cm^−2^, a frequency of 1000 Hz, and a 50% duty cycle. After deposition, the zinc coatings were treated by immersing them in a solution of polypropylene in dimethylbenzene at 130 °C for 30 min, then rinsed and dried, producing superhydrophobic composite layers. The water contact angle on untreated zinc coatings was 120 °, which increased to 170 ° after the polypropylene layer was applied. This superhydrophobicity stems from the combined hydrophobic properties of polypropylene and the porous zinc structure. Findings indicated that the superhydrophobic zinc coating significantly enhanced corrosion resistance. Polarization measurements showed that unmodified zinc coatings had a corrosion potential (*E*_corr_) of −1.072 V and a corrosion current density (*j*_corr_) (readers should note that different literature sources exhibit some inconsistency in the designation of corrosion current density, using either the letter i or j. According to IUPAC recommendations, j is the preferred symbol. However, in some borrowed figures and tables, the original author designations (i) have been preserved) of 2.53 × 10^−4^ A cm^−2^, whereas polypropylene-modified coatings exhibit improved resistance, with *E*_corr_ of −0.934 V and *j*_corr_ of 6.92 × 10^−5^ A cm^−2^ [25]. It should be observed that a superhydrophobic treatment by applying polymer films can also be performed on zinc coatings obtained from aqueous solutions. However, the use of DES-based zinc-plating baths significantly enhances the corrosion protection properties, primarily due to the formation of a specific porous structure in the zinc layer, which ensures strong adhesion of the polymer layer to the metallic substrate.

The impact of both inorganic and organic additives on zinc electrodeposition from deep eutectic solvents has been explored in some studies [26,27]. Abbott et al. [26] examined the effects of acetonitrile, aqueous ammonia, and ethylenediamine on zinc electrodeposition from electrolytes composed of choline chloride combined with either ethylene glycol or urea. It was shown that ammonia and ethylenediamine function as effective leveling and brightening agents, which notably influence the surface morphology of the zinc deposit and thereby enhance the corrosion resistance of coatings produced in aqueous baths. Similarly, Alesary et al. [27] investigated the roles of nicotinic acid, boric acid, and benzoquinone as additives in an ethaline-based zinc deposition solution, finding these substances to be effective brighteners that resulted in highly uniform and smooth zinc coatings.

Despite these advancements, the current literature indicates that the corrosion behavior of “pure” zinc deposits obtained from DES-based plating baths remains largely unexamined.

Since zinc coatings themselves generally exhibit low resistance in corrosive environments, traditional electroplating methods often incorporate alloying with other metals to improve their corrosion resistance [20].

**Figure 2:**
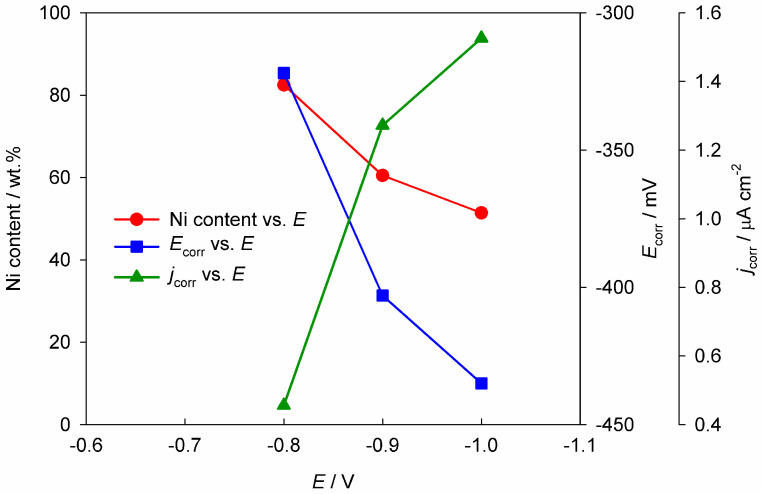
Corrosion potential (*E*_corr_) and corrosion current density (*j*_corr_) in 0.35 M NaCl solution for the Zn–Ni coatings deposited at different electrode potentials (0.45 M Zn(II); 0.05 M Ni(II) at 70 °C). The figure is plotted based on data from reference [28].

The electrodeposition of Zn–Ni alloys using DES-assisted electrolytes has been widely studied [28,29,30,31,32,33]. Fashu et al. [28] explored the deposition of a Zn–Ni alloy from a reline-based electrolyte across varying metal ion concentrations. Metal ions were added as chlorides at concentrations of 0.4 M Zn(II) and 0.1 M Ni(II); 0.45 M Zn(II) and 0.05 M Ni(II); and 0.475 M Zn(II) and 0.025 M Ni(II). Results showed that the relatively close electroreduction potentials of Ni(II) and Zn(II) ions enable their simultaneous deposition within the alloy. Depending on deposition conditions, the coatings incorporated between 10 and 50% zinc. An increase in Zn content was achievable by lowering the Ni(II) concentration, raising the temperature, and increasing cathodic polarization. The corrosion resistance of the coatings was influenced by their composition and surface morphology, with higher nickel content generally enhancing corrosion resistance (Figure 2). The most corrosion-resistant coating was obtained at 55 °C and a cathodic potential of −0.8 V from an electrolyte with 0.45 M Zn(II) and 0.05 M Ni(II), yielding a uniform, dense, and defect-free morphology due to an optimal alloy composition.

Li et al. [29] investigated the electrodeposition of Zn–Ni alloys from a reline-based solution with varying water contents (0, 1, 3, 5, and 7 wt.%). Introducing the controlled content of water into the electrolyte allowed for an adjustment of the composition of coatings, where both H_2_O concentration and deposition potential influenced the nickel content in an alloy, ranging from 4 to 96 wt.%. In a 3.5 wt.% NaCl solution, all Zn–Ni coatings demonstrated lower corrosion currents and more positive corrosion potentials compared to the steel substrate. Higher nickel content corresponded to improved corrosion resistance. Coatings containing significant nickel content exhibited more positive corrosion potential than steel when the water content in electrolytes was kept below 3 wt.%. Above this threshold, coatings with increased zinc content displayed slightly negative corrosion potentials relative to steel yet retained protective properties. The Zn–Ni alloy coating with approximately 14 wt.% Ni (γ-phase) offered the highest corrosion protection for steel in the NaCl environment.

**Figure 3:**
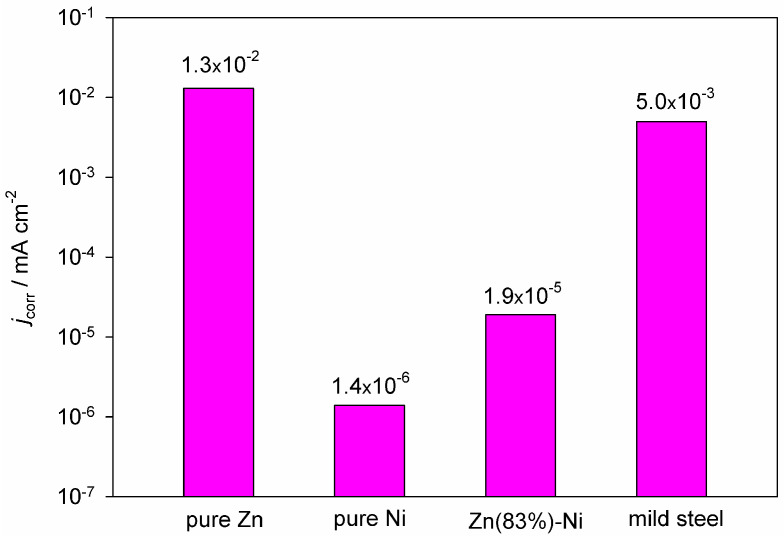
Corrosion current density of Zn, Ni, Zn–Ni coatings, and mild steel (a substrate) in 3.5% NaCl. The figure is plotted based on data from reference [30].

Lei et al. [30] studied the deposition of a Zn–Ni alloy coating from an ethaline-based electrolyte, which was modified by incorporating propylene carbonate to decrease the viscosity of the solution. Additionally, boric acid was included to enhance the surface morphology and adhesion of the coatings. This proposed electrolyte formulation enables the production of zinc–nickel alloy coatings (γ-phase) with a zinc content of approximately 81–85%, utilizing either potentiostatic or pulsed electrolysis techniques. The resulting coatings demonstrate excellent corrosion protection for the steel substrate in a corrosive 3.5% NaCl environment (Figure 3).

A comparative study was performed on the deposition processes of Zn–Ni alloy from electrolytes where the solvent was either a eutectic mixture of choline chloride and ethylene glycol (ethaline) or pure ethylene glycol [31]. The results revealed that increasing the concentration of ethylene glycol causes a shift in the deposition potentials of both nickel and zinc toward more negative values, suggesting the formation of corresponding nickel and zinc complexes in the solution. As the concentration of ethylene glycol is raised (transitioning from ethaline to pure ethylene glycol), the difference between the deposition potentials of the alloy components diminishes. The electrodeposition of the Zn–Ni alloy from both types of electrolytes follows the principles of anomalous co-deposition, where the more electronegative component, zinc, is preferentially deposited in the alloy. Coatings with nickel content ranging from 10 to 20 wt.% can be achieved from both electrolytes, which is particularly relevant for industrial applications in corrosion protection. Concurrently, a metastable γ-phase forms on the cathode, composed of nanocrystallites with average sizes of approximately 17.4 nm and 11.06 nm for the ethaline and pure ethylene glycol-based electrolytes, respectively. Table 2 summarizes results from the corrosion measurements. The corrosion potential of the Zn–Ni alloy is more negative than that of steel but slightly higher than that of pure zinc. This indicates that during electrochemical corrosion, the zinc component of the alloy primarily dissolves, resulting in the formation of a surface layer enriched in nickel, which acts as a physical barrier to further corrosion [31].

Alesary et al. [33] explored the influence of various additives, including boric acid, ammonium chloride, and nicotinic acid, on the electrochemical deposition of Zn–Sn alloy from an ethaline-assisted plating bath. In the absence of additives, the resulting coatings were dark gray and of poor quality. However, when boric and nicotinic acids were introduced, they facilitated the formation of uniform and bright deposits by adsorbing onto the cathode and suppressing the deposition processes of both zinc and tin. The addition of ammonium chloride was found to promote the formation of complexes with zinc ions, thereby inhibiting the partial deposition of zinc. These additives significantly improved the morphology, microstructure, and corrosion resistance of the coatings. The experimentally measured corrosion rates (mm year^−1^) were determined to be 0.0117 for pure zinc, 0.00369 for pure tin, 0.095 for the zinc–tin alloy without additives, 0.0609 for the zinc–tin alloy with boric acid, 0.0305 for the zinc–tin alloy with ammonium chloride, and 0.0875 for the zinc–tin alloy with nicotinic acid.

Bučko et al. [34] investigated the electrochemical deposition of a zinc–manganese alloy using ethaline as the solvent. Their findings indicated that the electrodeposited Zn–Mn coatings displayed excellent corrosion resistance in a 3% NaCl solution.

Electrolytes based on DESs can be utilized to produce not only corrosion-resistant zinc alloys but also composite coatings, which consist of an electrodeposited metal matrix embedded with particles of a solid dispersed phase [35]. For instance, Marín-Sánchez et al. [36] reported the electrodeposition of Zn/Ce_2_O_3_–CeO_2_ composite coatings from an electrolyte containing 0.3 M ZnCl_2_ and 0.3 M CeCl_3_·7H_2_O in reline. By varying the cathode deposition density from 1.13 to 0.75 A dm^−2^, they achieved coatings with a total cerium content ranging from 8.30 to 1.36 at.%, containing cerium oxides at different Ce oxidation states (approximately 50.6% Ce_2_O_3_ and 49.4% CeO_2_). The incorporation of Ce_2_O_3_ and CeO_2_ particles into the zinc matrix enhances the corrosion resistance of the coatings. Specifically, the corrosion rate of a zinc coating in an aggressive environment of 0.05 M NaCl is 2.29 × 10^−5^ A cm^−2^, whereas the corrosion rate decreases to a range of (1.62–1.78) × 10^−5^ A cm^−2^ when cerium oxide particles are included in the coating.

It is important to mention that the literature also reports on the electrodeposition of various zinc alloys from DES-based electrolytes, including Zn–Co [37] and Zn–Ti [38]. While it can be postulated that these coatings may exhibit enhanced corrosion resistance and protective characteristics, corresponding experimental studies have yet to be carried out.

Consequently, the application of DESs facilitates a broader range of chemical compositions for different types of zinc alloys compared to traditional aqueous electrolytes. However, there is currently a significant gap in research regarding the corrosion behavior of these coatings, underscoring the necessity for further investigation in this field as a pressing priority for future studies.

### 2.2. Corrosion Resistance and Protective Properties of Nickel-Based Coatings

Electrochemical deposition of nickel, along with coatings derived from its alloys and composites, represents one of the most ancient and widely utilized types of electroplating [39,40,41,42]. Nickel coatings are employed primarily for their corrosion-resistant and decorative features. Although nickel is a highly electronegative metal (exhibiting a standard potential of −0.25 V for the Ni(II)/Ni electrochemical couple), it tends to passivate, leading to a relatively positive potential and significant resistance to atmospheric exposure, alkaline environments, and certain acids. However, nickel is susceptible to rapid corrosion in various mineral acids. Within the nickel–iron galvanic corrosion couple, nickel serves as the cathode, offering protection to steel surfaces from corrosion only when the coating is free from pores or cracks. Typically, nickel is applied to steel with an intermediary copper layer, and the practice of electrodepositing multiple layers of nickel containing sulfur is common. The electrodeposition of nickel alloys not only enhances corrosion resistance but also improves other functional attributes, including hardness, wear resistance, magnetic characteristics, and electrocatalytic performance [42,43,44].

In industrial applications, nickel coatings are mainly deposited using aqueous electrolytes. Nevertheless, recent studies have investigated the electrodeposition of nickel, along with its alloys and composites, from solutions based on deep eutectic solvents [43,44,45,46,47,48,49,50,51,52]. It is important to highlight that a considerable number of published scientific articles in this area concentrate on the electrodeposition of nickel-based coatings. The following sections will outline only those studies that have examined the corrosion behavior and protective properties of the resulting coatings.

Nanocrystalline nickel coatings were successfully electrodeposited onto a brass substrate using an ethaline-based electrolyte at room temperature under potentiostatic conditions [46]. The coatings comprised Ni nanocrystals arranged in a face-centered cubic lattice, with an average crystallite size of about 6 nm. The findings indicated that these electrodeposits exhibited enhanced microhardness and notable corrosion resistance in a harsh environment containing 3% NaCl. Specifically, the corrosion potential for the brass substrate was approximately −220 mV, with a corrosion current density of 4.92 × 10^−4^ mA cm^−2^. In contrast, the corresponding values for the nanocrystalline nickel coating were around −690 mV and 11.02 × 10^−2^ mA cm^−2^. Consequently, the nickel layers provided sacrificial anode protection for the brass substrate in a 3 wt.% NaCl aqueous solution at room temperature.

Utilizing an ethaline-based electrolyte presents extensive opportunities for tailoring the micro/nanoarchitecture of electrodeposited nickel layers through controlled electrolysis techniques [47]. For example, during stationary potentiostatic electrolysis, nanoplates measuring 10–50 nm can be generated on the surface, with edges that resemble arrowheads. In pulsed mode, depending on the duty cycle, coatings can exhibit a surface morphology akin to fingerprints, arranged in a series of narrow strips and nanoplates. When employing reverse pulse mode, flower-like clusters with a hierarchical structure are formed on the surface. The nanostructuring of the surface contributes to the development of superhydrophobic properties in the nickel layers, which significantly enhances their corrosion resistance in a 3 wt.% NaCl aqueous solution. Specifically, the corrosion potential of the brass substrate was approximately −0.225 V (vs. the saturated calomel electrode), while the superhydrophobic nickel layer exhibited a corrosion potential of −0.710 V, notably more negative than that of the substrate. Thus, nickel coatings deposited from the DES-based electrolyte offer effective corrosion protection for brass surfaces.

Bernasconi and Magagnin [49] studied the electrodeposition of nickel onto aluminum surfaces using a deep eutectic solvent. Their findings revealed that the deposits produced from the DES-based electrolyte exhibited lower corrosion resistance, rendering them unsuitable for industrial applications. However, the corrosion resistance significantly improved when a nickel intermediate layer was first deposited from the DES-based electrolyte on the aluminum surface, followed by the application of a final layer from an aqueous nickel-plating solution. This approach suggests that the DES-assisted plating electrolyte effectively forms a well-adhered layer on the aluminum alloy’s surface without passivating the substrate. Consequently, it is recommended to deposit nickel from aqueous plating baths onto this intermediate layer.

Research has demonstrated that incorporating water into deep eutectic solvents can significantly enhance several physicochemical and operational parameters of the nickel electrodeposition process [50,51]. However, water should not be viewed merely as an additional solvent; rather, it serves as an extra source of hydrogen bonds in the formation of the deep eutectic solvent [51]. When added within certain concentration limits, the electrolyte based on a DES maintains a specific hole mechanism for mass and charge transfer, effectively remaining an ionic liquid without transforming into a highly concentrated aqueous solution.

**Figure 4:**
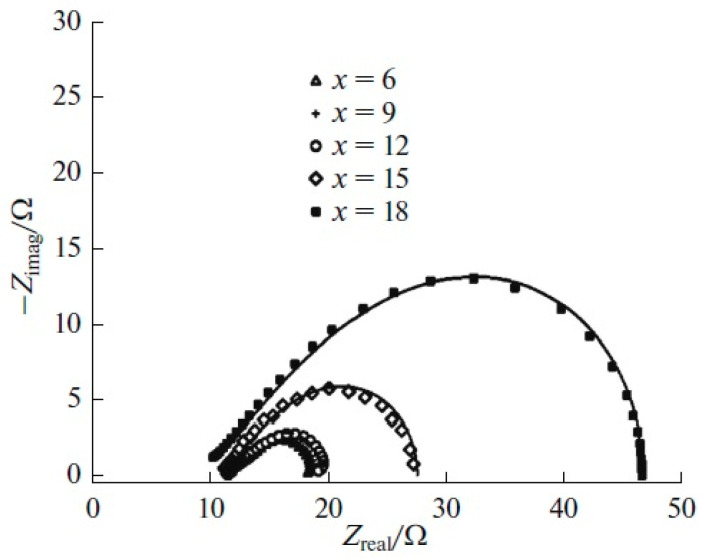
Nyquist diagrams of nickel coatings electrodeposited from electrolytes containing ethaline + NiCl_2_ + *x*H_2_O. Symbols represent experimental values and solid lines reflect calculated results. Reprinted from [50] with permission from Springer Nature, copyright 2017.

**Figure 5:**
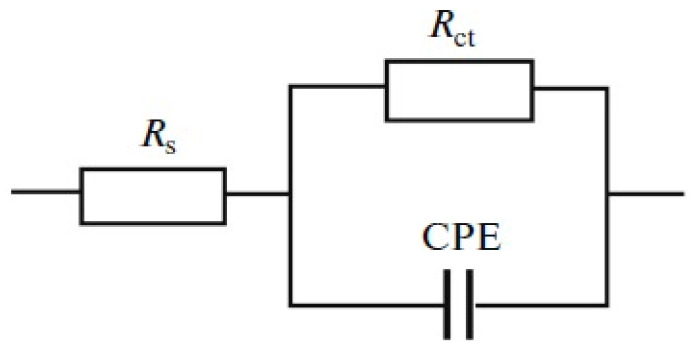
Equivalent circuit simulating the electrochemical impedance of the corroding electrode/solution surface. Reprinted from [50] with permission from Springer Nature, copyright 2017.

The addition of extra water to nickel-plating electrolytes based on deep eutectic solvents results in reduced viscosity and enhanced electrical conductivity, which is beneficial for the potential practical application of these systems [51]. Moreover, incorporating water into the electrolyte composition enables control over the microstructure and various properties of the resulting coatings [50]. Nanocrystalline coatings with a face-centered cubic lattice are deposited from nickel-plating electrolytes based on ethaline with water additives, with average crystallite sizes ranging from 3.1 to 7.2 nm. The inclusion of additional water in the DES-based nickel-plating electrolytes leads to increased microhardness of the deposits and improved corrosion resistance. Figure 4 presents Nyquist diagrams for the corrosion of nickel coatings in a 0.05 M H_2_SO_4_ solution. These impedance plots take the form of compressed semicircles (it should be noted that the Nyquist plot in Figure 4 are not presented on an orthonormal scale), with their centers located below the real axis, indicating that the corrosion rate of nickel coatings is governed by charge transfer at the heterogeneous electrode surface, which can be described by a constant phase element (CPE) model [53]. To analyze the electrochemical impedance measurement results, the equivalent circuit depicted in Figure 5 was utilized. This equivalent circuit includes the polarization resistance of the electrode reaction (*R*_ct_), the constant phase element characterized by two parameters *Q* and *n*, and the ohmic resistance of the solution (*R*_s_). The calculated kinetic parameters are summarized in Table 3.

An increase in the polarization resistance *R*_ct_ with higher water content in the nickel-plating electrolyte indicates improved corrosion resistance of the nickel deposits. Concurrently, the value of *Q* decreases while the parameter *n* increases. Analyzing the obtained data suggests that adding extra water to the NiCl_2_·6H_2_O solution in ethaline reduces the roughness and heterogeneity of the nickel coatings, thereby enhancing their corrosion resistance in a 0.05 M H_2_SO_4_ solution [50]. It is suggested that water molecules in the nickel-plating electrolyte are adsorbed onto the coating and interact with the active sites (such as crystal defects) of the metal, effectively blocking them. This process facilitates the rapid formation of a continuous and stable passivating nickel hydroxide film. Additionally, the corrosion resistance of the coatings is further enhanced due to surface leveling, as evidenced by the increased values of the parameter *n*.

Incorporating a secondary component into the nickel matrix can enhance the corrosion resistance and protective qualities of the resulting coatings by forming an alloy with nickel [40,42]. Wang et al. [54] documented the synthesis of a nickel–copper binary alloy from a reline-based electrolyte containing 0.6 M NiCl_2_·6H_2_O and 0.01 M CuCl_2_·H_2_O. Unlike conventional aqueous systems, the deposition potentials for copper and nickel in DESs are nearly identical, enabling alloy formation without additional additives. By adjusting the cathodic current density, the copper content in the alloy can be varied, resulting in compositions with copper content from 5 to 40 at.%. Polarization assessments in a 3.5 wt.% NaCl solution revealed that Ni–Cu alloy coatings (approximately 17.6 at.% Cu), electrodeposited from the reline-based electrolyte, displayed superior corrosion resistance compared to both a commercial Monel-400 alloy (~28 at.% Cu) and an alloy with a higher copper content (~33.6 at.%) deposited from an aqueous solution. The corrosion current densities for these coatings were reported to be 3.17 × 10^−7^, 2.24 × 10^−6^, and 1.27 × 10^−6^ A cm^−2^, respectively [54].

**Figure 6:**
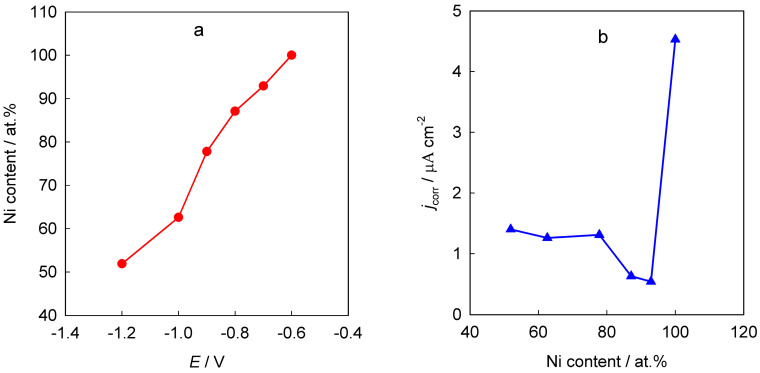
Dependences of the nickel content in the Ni–Zn coating on the electrodeposition potential (**a**) and the corrosion current density in 0.1 M NaCl on the nickel content in the coating (**b**) for Ni–Zn coatings deposited from an electrolyte containing 0.1 M NiCl_2_ and 0.4 M ZnCl_2_ in reline at 70 °C. The figure is plotted based on data from reference [55].

Yang et al. [55] examined the electrolytic deposition of a Ni–Zn alloy using an electrolyte composed of 0.1 M NiCl_2_ and 0.4 M ZnCl_2_ dissolved in reline. Their findings demonstrated that nickel, being the more electropositive metal, tends to deposit preferentially over zinc. Zinc deposition occurs only at sufficiently negative potentials, thus limiting its concentration in the coating to below 50 at.% (Figure 6a). The resulting Ni–Zn alloy formed a solid solution. Coatings with nickel content at or above 87 at.% exhibited enhanced corrosion resistance (Figure 6b) attributed to their dense nonporous microstructure.

You et al. [56] explored the electrodeposition of nickel–cobalt alloys from ethaline-based electrolytes, observing that higher cobalt ion concentrations in the electrolyte produce coatings with cobalt content ranging from 4% to 40%. However, the presence of cobalt in these coatings results in elevated corrosion currents when exposed to a 3% NaCl aggressive environment. For instance, corrosion current densities of 2.5, 3.7, 5.8, and 6.1 μA cm^−2^ were recorded for coatings with cobalt content of 0, 4, 18, and 40 wt.%, respectively. In contrast, Li et al. [57] investigated Ni–Co alloys deposited from a similar ethaline-based solution and found that increasing cobalt content tended to reduce corrosion current density in a 3.5% NaCl solution. These differing findings on the corrosion behavior of Ni–Co electrodeposited alloys indicate a need for further investigation to clarify their protective performance.

**Figure 7:**
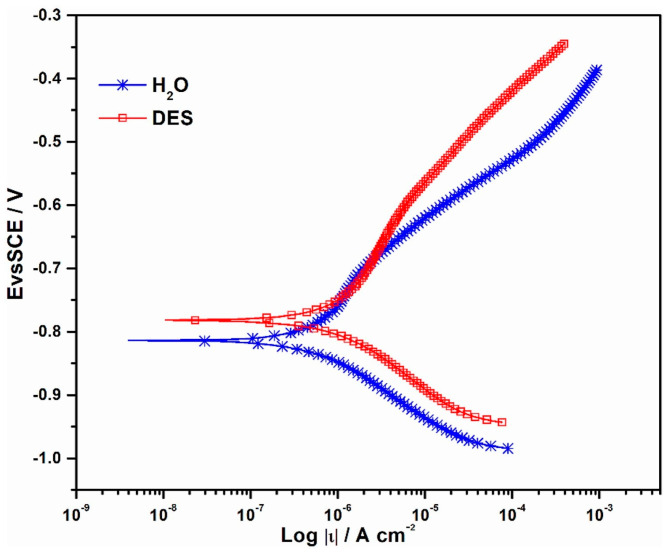
Polarization curves characterizing the corrosion behavior of Ni–Co alloy coatings in an aqueous 3 wt.% NaCl solution. H_2_O—coating deposited from a conventional aqueous solution; DES—coating deposited from ethaline. Reprinted from [58] under Creative Commons Attribution License (CC BY).

A detailed comparative study was also conducted on the electrochemical deposition patterns of nickel–cobalt alloy from a DES (ethaline) and a conventional aqueous electrolyte [58]. Although the kinetics of cathodic processes differ significantly between these two types of electrolytes, they produce alloys with similar nickel and cobalt content. It was found that specific surface morphology features lead to some variations in the corrosion resistance of the alloy deposited from DESs (Figure 7); the corrosion potential is −0.785 V compared to −0.812 V for the coating from an aqueous solution, and the corrosion current density is 0.927 μA cm^−2^, which is higher than 0.336 μA cm^−2^ for the coating derived from an aqueous solution. According to [58], these less encouraging results suggest that the optimal composition of DES-based plating baths for depositing corrosion-resistant Ni–Co alloy coatings remains to be refined in future research.

Vijayakumar et al. [59] investigated the electrodeposition of a ternary Ni–Co–Sn alloy using an ethaline-based electrolyte, alongside Ni–Sn and Co–Sn binary alloys as references. Their findings highlighted the potential of the Ni–Co–Sn alloy as an electrocatalyst for hydrogen evolution in alkaline solutions, demonstrating both higher current density for the hydrogen evolution reaction and superior corrosion resistance compared to the binary alloys in a 1 M KOH environment. The measured corrosion current densities for Ni–Sn, Co–Sn, and Ni–Co–Sn alloys were 1.167 × 10^−3^, 1.472 × 10^−3^, and 5.340 × 10^−4^ A cm^−2^, respectively, indicating the enhanced stability and catalytic efficiency of the ternary alloy.

Nanocrystalline nickel–molybdenum (Ni–Mo) alloys have been effectively electrodeposited from a reline-based electrolyte [60]. The molybdenum content in these coatings ranged from 2 to 35 wt.% depending on electrolysis parameters and electrolyte composition. Adding molybdenum to nickel significantly improved the corrosion resistance of the coatings. In a 0.5 M NaCl solution, the corrosion current density for pure nickel was 169 μA cm^−2^, whereas for a Ni–Mo alloy containing 8–9 wt.% Mo, it was only 3.94 μA cm^−2^. Notably, Ni–Mo alloys produced from DESs demonstrated enhanced corrosion resistance compared to similar coatings from conventional aqueous electrolytes. This improvement was linked to the distinctive surface morphology and nanocrystalline structure of coatings deposited from DESs, characterized by greater uniformity and fewer defects. High corrosion resistance was also observed in Ni–Mo coatings produced from an electrolyte based on a eutectic mixture of choline chloride and propylene glycol [43].

**Figure 8:**
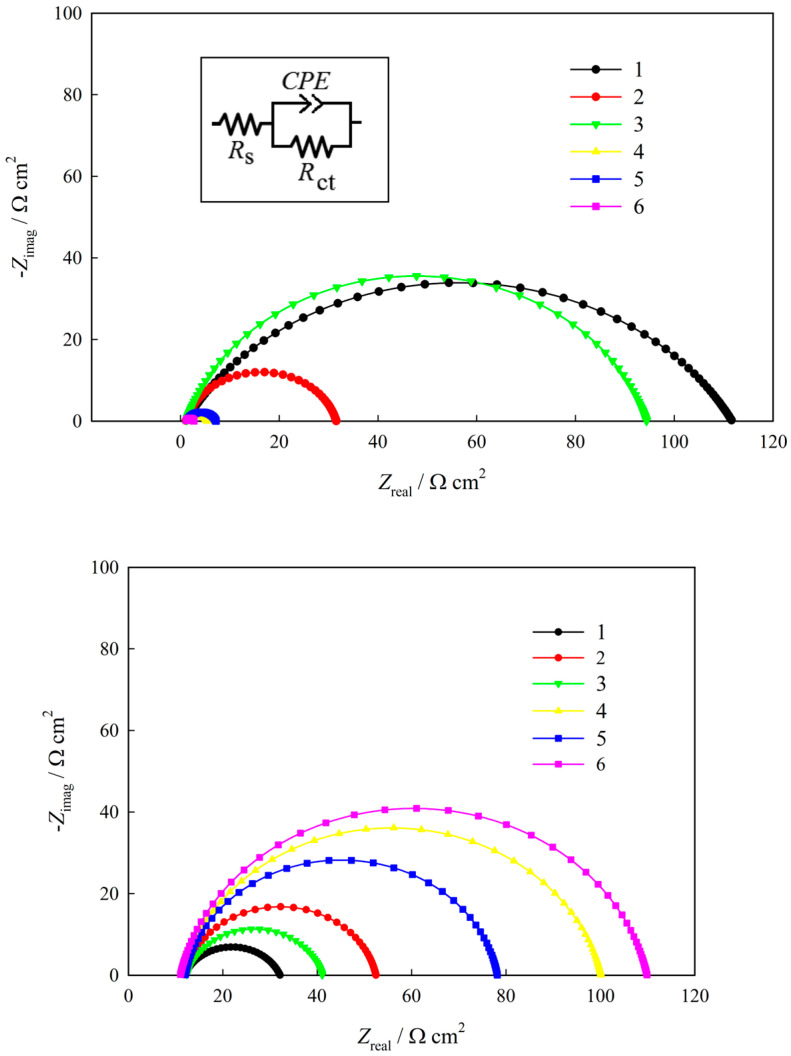
Nyquist plots for hydrogen evolution in 1 M NaOH (**above**) and for electrochemical corrosion in 0.05 M H_2_SO_4_ (**below**) of the Ni–La layers deposited from an ethaline-based solution. The numbering of the curves corresponds to that given in Table 4 (Reproduced from [61] with permission from Elsevier).

The results of a recent study [61] demonstrated that the micromodification of DES-electrodeposited nickel by introducing lanthanum into its chemical composition (up to 1.75 wt.%) significantly enhances both the electrocatalytic activity of the Ni–La alloy in the hydrogen evolution reaction in an alkaline medium and the corrosion resistance of the coating in an aggressive 0.05 M H_2_SO_4_ environment. These findings were supported by electrochemical impedance spectroscopy measurements and their interpretations (Figure 8, Table 4). Such positive effects were primarily attributed to the formation of lanthanum-containing active sites on the surface. Additionally, the improvement in corrosion resistance could be linked to the formation of a denser and more compact passive layer on the Ni–La alloy surface and the reduction in grain size. Thus, Ni–La alloy coatings deposited from DESs may hold potential applications as corrosion-resistant electrocatalytic materials for green hydrogen energy systems [61].

**Figure 9:**
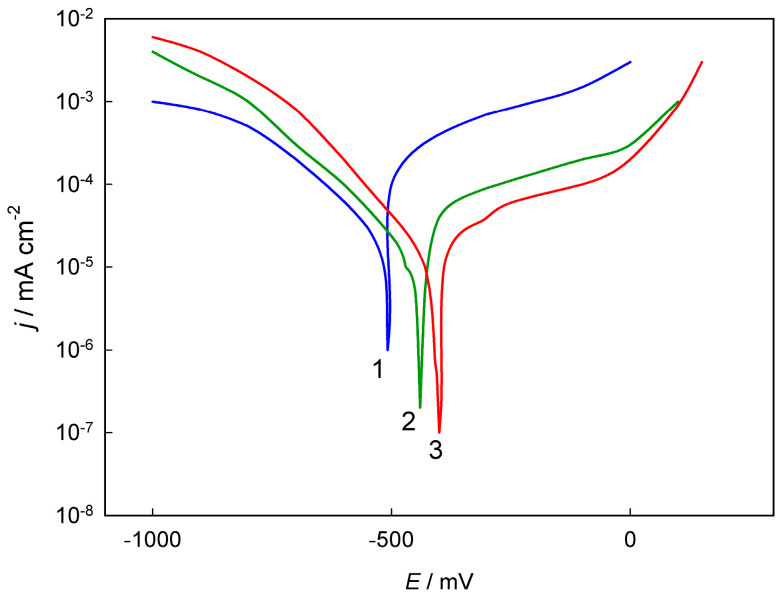
Polarization curves of nickel (1), nickel–titania (5%) (2), and nickel–titania (10%) (3) electrodeposits in 3% NaCl. The sweep rate was 0.001 V s^−1^. Reprinted from [64] under Creative Commons Attribution License (CC BY).

Deep eutectic solvent-based electrolytes are also applicable for the electrochemical deposition of composite coatings with a nickel matrix. Incorporating a dispersed phase within the nickel matrix has been shown to notably enhance the corrosion resistance of the resulting coatings [62,63,64]. For example, Figure 9 illustrates polarization curves for the electrochemical corrosion behavior of pure Ni and Ni–TiO_2_ coatings in a 3% NaCl corrosive environment, with calculated corrosion parameters summarized in Table 5 [64]. The data indicate that introducing titania nanoparticles into the nickel matrix and increasing their concentration results in a positive shift in corrosion potential and a reduction in corrosion current density. This enhanced corrosion resistance is attributed to the barrier effect (a partial shielding of the metal matrix from the contact with aggressive medium), the formation of numerous microcells that mitigate localized corrosion, and the elongation of current paths in the corrosion process, which further inhibits electrochemical degradation [65].

Thus, utilizing DES-based plating baths allows for a broader variety of nickel alloys and composites to be produced, with precise control over the coating composition by adjusting electrolysis parameters. This approach offers enhanced opportunities to improve corrosion resistance relative to traditional aqueous systems.

### 2.3. Corrosion Resistance and Protective Properties of Chromium-Based Coatings

Electrochemical chromium plating is a highly prevalent method of coating deposition due to its unique multifunctional properties [39,66]. Chromium coatings are widely used largely because of their good surface appearance and a unique set of excellent service properties. Although the electrochemical couple Cr(III)/Cr has a standard potential of −0.74 V, which is relatively negative, chromium’s tendency to passivate leads to the formation of a thin dense oxide layer that effectively resists corrosion in a variety of aggressive environments. These coatings are commonly applied for hard, wear-resistant, and anti-friction purposes, retaining their attractive appearance over extended periods [67].

A significant drawback of traditional chromium plating processes is the use of concentrated solutions of hexavalent chromium compounds, which are highly toxic and hazardous. Given this, the use of hexavalent chromium is now significantly restricted [68]. Environmentally friendly chromium plating baths utilizing trivalent chromium (Cr(III)) salts present a viable alternative to those based on Cr(VI) compounds [69]. However, advancements in this area have been relatively slow, primarily due to the complex chemistry and electrochemical behavior of trivalent chromium compounds in aqueous solutions [70,71].

In this context, utilizing deep eutectic solvent-assisted trivalent chromium plating baths presents a promising alternative to traditional water-based electrolytes for chromium plating [72,73,74,75,76,77,78,79]. These DES-based systems offer several advantages over their aqueous counterparts, such as enhanced environmental safety, reduced wastewater generation, stability in chemical composition, the capability to deposit a variety of chromium-based alloys and composites, and improved current efficiency. Additionally, chromium coatings produced from DES-based electrolytes demonstrate increased electrocatalytic activity [80], which can be attributed to their distinctive electrochemical properties and specific corrosion behaviors that have been explored in a number of studies.

**Figure 10:**
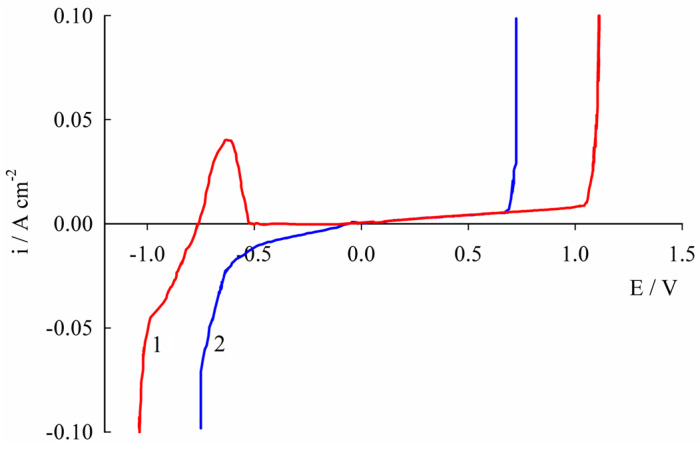
Polarization dependences of deposits in 0.5 M H_2_SO_4_ solution at 25 °C: (1) coatings from hexavalent chromium bath (250 g dm^−3^ CrO_3_ + 2.5 g dm^−3^ H_2_SO_4_) at 40 A dm^−2^ and 45 °C; (2) coatings from electrolyte containing CrCl_3_ + 2.5ChCl + 15H_2_O at 5 A dm^−2^ and 40 °C. Scan rate is 50 mV s^−1^. Used with permission of Emerald Publishing Limited from [76]; permission conveyed through Copyright Clearance Center, Inc.

Protsenko et al. [76] investigated the electrodeposition of coatings from a deep eutectic solvent formulated as 1CrCl_3_ + 2.5ChCl + 15H_2_O, where the numerical values denote molar ratios. The resulting coatings exhibited an amorphous microstructure and contained elements such as chromium, carbon, oxygen, and chlorine. Notably, metalloids in the coatings originate from the corresponding components of the electrolyte. The incorporation of carbon into coatings derived from Cr(III) electroplating baths is a common phenomenon, facilitated through either a chemical or electrochemical mechanism [81]. This altered chemical composition, coupled with the formation of an amorphous structure, significantly influences the electrochemical corrosion behavior of the coatings. For instance, the polarization curve representing the anodic dissolution of “conventional” chromium in an acidic environment reveals the classic phases of active dissolution, passive state, and transpassive dissolution (Figure 10, curve 1). In contrast, coatings obtained from the DES-based electrolyte did not exhibit a segment of active dissolution; instead, the segment of a polarization curve associated with cathodic hydrogen evolution shifted toward more positive potentials (Figure 10, curve 2) [76]. This behavior indicates a high corrosion resistance of the coatings produced from the DES, which is attributed to the development of a particular surface protective film enriched with carbon [82]. Carbon and its compounds with chromium, such as chromium carbides, function as specific “cathodic agents” that effectively shift the corrosion potential into the passive state region, thereby significantly mitigating the rate of corrosion degradation.

**Figure 11:**
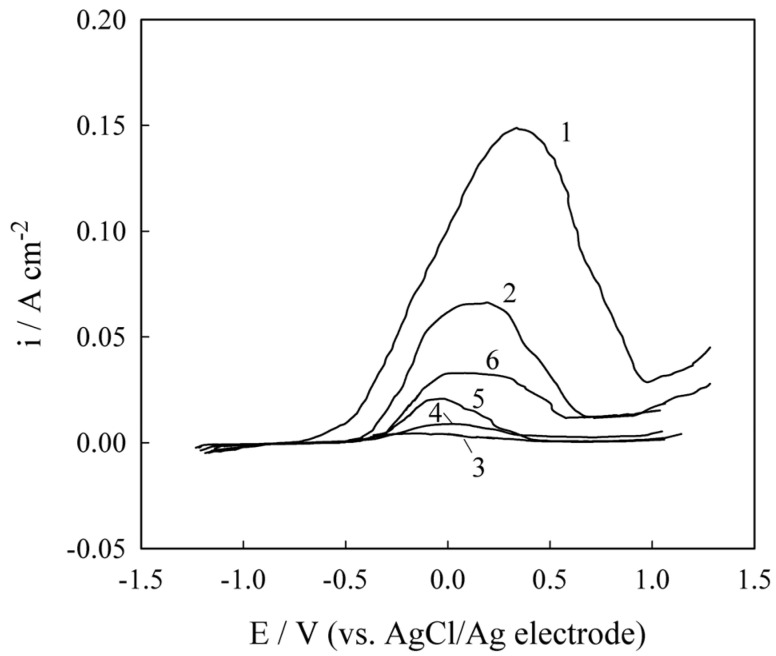
Polarization curves of a steel sample (1) and steel samples coated with chromium layers with a thickness of 2.5 μm (2), 5 μm (3), 10 μm (4), 15 μm (5), and 20 μm (6) recorded in 0.1 N aqueous Na_2_SO_4_ solution (pH 3.0). Reprinted from [78] under Creative Commons Attribution License (CC BY).

A study was conducted to evaluate the corrosion resistance and protective properties of coatings deposited from a liquid mixture comprising choline chloride, chromium (III) chloride, and water, following a molar ratio of 2.5:1:12, respectively [78]. Coatings of varying thicknesses (2.5, 5, 10, 15, and 20 μm) were deposited on a steel substrate. A 0.1 N Na_2_SO_4_ solution with a pH of 3.0 served as the aggressive medium. In this acidic environment, chromium exists in a passive state, preventing it from undergoing active dissolution (Figure 11). Consequently, by disregarding the dissolution rate of chromium in comparison to that of iron (the steel substrate, which dissolves through pores and cracks in the coating), the degree of protection (*DP*) can be calculated using the following equation:

$$DP=\frac{j_{s}-j}{j_{s}}\cdot 100\%$$

where *j* is the maximum current density of the anodic dissolution of steel with a Cr deposit and *j*_s_ is the maximum current density of anodic dissolution of steel without a Cr deposit.

The results shown in Table 6 indicate that the degree of protection achieves its highest value of 97.5% when the coating thickness is increased to 5 μm. However, any further increase in thickness results in a gradual decline in protective properties. This reduction may be attributed to the cracking of the coatings and an increase in their defect density [78].

**Figure 12:**
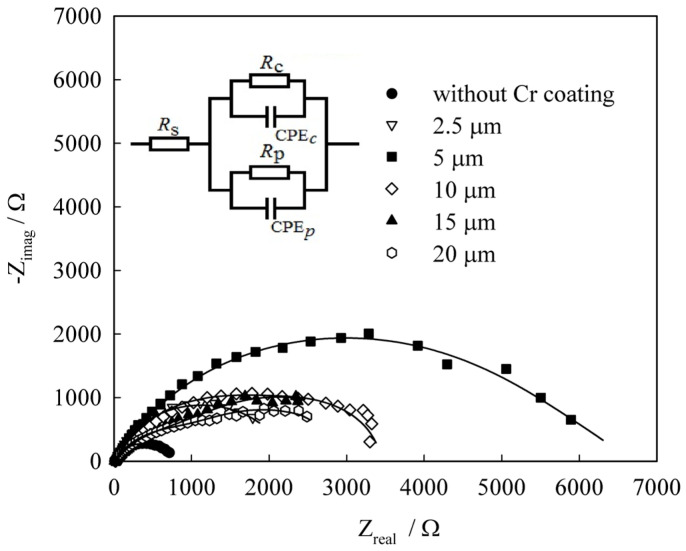
Nyquist plots of mild steel (1) and Cr deposits on mild steel substrate (2–6) in 0.1 N Na_2_SO_4_ (pH 3.0) at open circuit potential. The thickness of chromium deposits (μm) was 2.5; 5; 10; 15; and 20. The inset shows the equivalent circuit used to interpret the impedance of electrode/solution interface. Reprinted from [78] under Creative Commons Attribution License (CC BY).

The electrochemical impedance spectroscopy (EIS) technique was employed to conduct an in-depth investigation into the corrosion and protective properties of chromium coatings. The findings revealed that a coating thickness of 5 μm was associated with the highest polarization resistances for the electrode reaction (*R*_C(p)_), as well as the lowest values of capacitive components (*Q*_C(p)_) and dimensionless indices (*n*_C(p)_) in the constant phase element (Figure 12, Table 7). These results indicate superior protective and corrosion characteristics. This observation challenges the widely held belief that thicker chromium coatings inherently offer better corrosion protection. These insights are crucial for the practical application of chromium coatings deposited from deep eutectic solvents.

Several studies have been published focusing on the electrochemical deposition of chromium alloys with various metals and metalloids from deep eutectic solvents, examining the corrosion characteristics of the resulting deposits [83,84,85,86,87].

Chromium–phosphorus coatings were successfully deposited from an ethaline-based electrolyte containing 0.3 M CrCl_3_·6H_2_O, 0.2 M NaCl, and 0.05 M NH_4_H_2_PO_2_ [83]. The resulting coatings included approximately 6 wt.% phosphorus, along with trace amounts of Cr_2_O_3_ and Cr(OH)_3_, in addition to the primary metal component. It was observed that the corrosion resistance of the Cr–P alloy coatings exceeded that of “pure” chromium in a corrosive environment of 0.1 M H_2_SO_4_; however, this superiority diminished in the presence of 3.5 wt.% NaCl, where the corrosion resistance was lower.

Nanocrystalline coatings of an Fe–Cr alloy were electrodeposited from an ethaline-based deep eutectic solvent without the addition of any organic components [84]. The resulting coatings exhibited high corrosion resistance in a 5 wt.% NaCl aqueous solution. This enhanced resistance was attributed to the nanocrystalline structure of the coating, characterized by an average particle size of approximately 1.53 nm, as well as the formation of a highly compact protective passive film consisting of Cr_2_O_3_ and Fe_2_O_3_ oxides. Similar findings have been reported in another study [85].

Saravanan and Mohan [86] demonstrated the feasibility of electrodepositing a ternary chromium–nickel–iron alloy, containing 4–15% Cr, 34–41% Ni, and 53–61% Fe, from an ethaline-based electrolyte. Coatings with this composition can rival highly corrosion-resistant stainless steels. Potentiodynamic polarization tests and electrochemical impedance spectroscopy revealed that the deposited Fe_54.62_Ni_30.87_Cr_14.5_ alloy exhibits exceptional corrosion resistance in an aggressive 3.5% NaCl environment, outperforming that of mild steel substrates. It is noteworthy that the deposition of Fe–Cr–Ni ternary alloys from conventional aqueous solutions poses significant challenges [87]; therefore, the application of DES-based electrolytes offers promising opportunities in this area.

The application of DES-assisted plating baths for the electrochemical deposition of corrosion-resistant chromium-based composite coatings is still relatively underexplored in the literature. Nonetheless, one study [88] reports the fabrication of Cr–single-walled carbon nanotube (SWCNT) composite coatings from an ethaline-based solution utilizing pulse electrolysis. The incorporation of single-walled carbon nanotube particles led to a marked enhancement in both microhardness and corrosion resistance of the coatings when exposed to a 3.5 wt.% NaCl environment. Specifically, the corrosion current densities for the chromium coating and the Cr–SWCNT composite were found to be 312.7 and 180.9 μA cm^−2^, respectively.

## 3. Conclusions

The review of the literature indicates that electrolytes based on deep eutectic solvents offer an effective alternative for the electroplating of corrosion-resistant and protective coatings. Compared to conventional aqueous plating solutions, DES-based systems provide several advantages, including enhanced environmental compatibility, economic and technological benefits, and the ability to precisely control the chemical composition and microstructure of coatings. This control allows for coatings with superior physicochemical properties, such as greater corrosion resistance in corrosive environments and improved protective characteristics. DES-assisted electroplating enables the production of nanocrystalline alloys that incorporate nickel, zinc, chromium, and other metals or metalloids, with the ability to adjust the coating’s composition and properties across a wide range. Moreover, DESs hold significant potential for composite coatings, as they promote stability against particle aggregation and sedimentation, enabling the uniform incorporation of dispersed phases within metal matrices.

The advantages of DESs also extend to improved current efficiency and deposition rates, contributing to a more energy-efficient and economical electrodeposition process. These electrolytes have demonstrated compatibility with complex bath compositions, allowing for the integration of various alloying elements and particles, which can enhance wear resistance and mechanical durability in addition to corrosion protection. Furthermore, the relatively low volatility and toxicity of DESs represent critical benefits over traditional hexavalent chromium baths, especially in applications where environmental and health standards are stringent.

Despite these benefits, a lack of systematic studies addressing the electrochemical deposition of DES-based coatings and their corrosion resistance and protective properties presents challenges in fully assessing the potential of these systems for practical use. Continued research is therefore essential to establish reliable links between the composition, structure, and performance of these coatings and to refine synthesis and application methods. Investigating the scalability of DES-assisted deposition methods in industrial applications and understanding long-term performance under varied conditions will be particularly valuable. Such efforts will broaden the range of DES-based electroplating technologies and facilitate the development of advanced materials with specific properties suited to diverse industrial and technological applications.

Despite the promising advantages of DES-based electroplating systems, there are some limitations that need to be addressed in future research. One limitation is the relatively limited number of studies on the long-term stability and real-world performance of coatings produced using DESs, particularly under harsh environmental conditions. Furthermore, while DESs offer precise control over the composition and microstructure of coatings, the scalability of these processes for large-scale industrial applications has yet to be fully explored. Additionally, the full environmental impact of large-scale DES production and disposal remains unclear and requires further investigation. Another limitation is the current lack of standardization in the electroplating process using DESs, which can hinder reproducibility and comparison across different studies. Addressing these limitations will be essential for the successful integration of DES-based electroplating technologies in industrial applications.

## Figures and Tables

**Table 1:** Melting points of ethaline and reline and their individual constituents.

Substance or Eutectic Mixture	Melting Point, °C
ethylene glycol	−12.9
urea	134
choline chloride	303
DES ethaline (ethylene glycol + choline chloride)	−66
DES reline (urea + choline chloride)	12

**Table 2:** Corrosion data for Zn–Ni alloys and Fe substrate in 3.5% NaCl solution. Reprinted from [31] (copyright © 2020 American Chemical Society, under Creative Commons Attribution License (CC BY).

Sample	*E*_corr_ (mV vs. Ag/AgCl Reference Electrode)	*j*_corr_ (μA cm^−2^)
Fe substrate	−402	7.5
20% Ni deposited from ChCl/EG 1:2	−902	1.6
20% Ni deposited from ChCl/EG 1:4.5	−859	4.3
15% Ni deposited from ChCl/EG 1:4.5	−774	3.7
12% Ni deposited from ChCl/EG 1:4.5	−853	7.9
18% Ni deposited from pure EG	−852	18.1
15% Ni deposited from commercial ZnNi plating bath	−826	11.8

**Table 3:** Calculated parameters of the equivalent circuit that simulates the corrosion of nickel coatings in a solution of 0.05 M H_2_SO_4_ at the corrosion potential. Reprinted from Ref. [50] with permission from Springer Nature, copyright 2017.

System ^1^	*R*_s_, Ω	*R*_ct_, Ω	*Q*, Ω^−1^ s*^n^*	*n*
Ethaline + NiCl_2_·6H_2_O	11.37	8.45	78.81 × 10^−3^	0.526
Ethaline + NiCl_2_·9H_2_O	11.22	8.81	68.24 × 10^−3^	0.530
Ethaline + NiCl_2_·12H_2_O	11.49	8.85	51.04 × 10^−3^	0.629
Ethaline + NiCl_2_·15H_2_O	11.05	16.32	1.24 × 10^−3^	0.985
Ethaline + NiCl_2_·18H_2_O	10.61	22.83	84.61 × 10^−6^	0.991

^1^ The numbers before H_2_O in the first column indicate the molar amount of water.

**Table 4:** Simulation of electrochemical impedance spectra registered for hydrogen evolution and corrosion reactions (Reproduced from [61] with permission from Elsevier).

Sample Number	Content of La in Coating/wt.%	Parameters for Hydrogen Evolution				Parameters for Corrosion			
		*R*_s_/Ω	*R*_ct_/Ω cm^2^	*CPE*		*R*_s_/Ω	*R*_ct_/Ω cm^2^	*CPE*	
				*Y*/Ω^−1^ s*^n^* cm^−2^	*n*			*Y*/Ω^−1^ s*^n^* cm^−2^	*n*
1	–	1.05	110.65	6.80 × 10^−4^	0.70	11.85	20.28	17.80 × 10^−5^	0.76
2	0.65	1.15	30.40	8.80 × 10^−5^	0.85	11.90	40.55	7.80 × 10^−5^	0.88
3	0.48	1.20	93.18	7.48 × 10^−5^	0.83	12.35	28.73	8.95 × 10^−5^	0.85
4	1.51	1.80	3.60	6.95 × 10^−5^	0.79	11.45	88.70	6.12 × 10^−5^	0.87
5	1.41	1.35	5.84	7.82 × 10^−5^	0.81	12.05	66.12	6.44 × 10^−5^	0.90
6	1.75	1.15	1.55	5.67 × 10^−5^	0.87	11.00	98.87	6.06 × 10^−5^	0.88

**Table 5:** Corrosion potential and corrosion current density of Ni and Ni–TiO_2_ coatings in a 3% NaCl water solution extracted from Tafel analysis. Reprinted with modification from [64] under Creative Commons Attribution License (CC BY).

Coating	*E*_corr_ (mV)	*j*_corr_ (mA cm^−2^)
Ni	−508	9.31 × 10^−6^
Ni–TiO_2_ (5%)	−451	3.45 × 10^−6^
Ni–TiO_2_ (10%)	−408	1.28 × 10^−6^

**Table 6:** The effect of coating thickness on the degree of protection (DP). Reprinted from [78] under Creative Commons Attribution License (CC BY).

Chromium Deposits Thickness (μm)	*DP* (%)
2.5	55.8
5	97.5
10	94.1
15	86.4
20	78.3

**Table 7:** The calculated electrochemical impedance parameters of the corrosion of chromium coatings and steel substrate. Reprinted from [78] under Creative Commons Attribution License (CC BY).

Thickness of Deposit (μm)	*R*_s_ (Ω)	Characteristics of Corrosion of Cr Deposits			Characteristics of Corrosion of Steel Substrate Through Pores		
		*R*_c_ (Ω cm^2^)	*Q*_c_ × 10^6^ (Ω^−1^ s*^n^* cm^−2^)	*n*_c_	*R*_p_ (Ω cm^2^)	*Q*_p_ × 10^3^ (Ω^−1^ s*^n^* cm^−2^)	*n*_p_
– (steel substrate)	10.5	–	–	–	748	2010	0.650
2.5	10.5	940	1180	0.675	2199	4.09	0.700
5	10.0	5050	39	0.959	3595	0.73	0.998
10	10.3	2310	200	0.807	3100	2.5	0.997
15	10.4	1010	680	0.795	2500	2.9	0.800
20	10.2	950	780	0.755	2200	3.9	0.500

## Data Availability

The data presented in this study are available upon request from the corresponding author. The data are not publicly available due to technical limitations.
